# Steps Ahead: optimising physical activity in adults with cystic fibrosis: Study Protocol for a pilot randomised trial using wearable technology, goal setting and text message feedback.

**DOI:** 10.12688/hrbopenres.13025.3

**Published:** 2020-11-16

**Authors:** Maire Curran, Audrey C. Tierney, Louise Collins, Lauren Kennedy, Ciara McDonnell, Andrew J. Jurascheck, Ali Sheikhi, Cathal Walsh, Brenda Button, Rose Galvin, Brian Casserly, Roisin Cahalan

**Affiliations:** 1 School of Allied Health, University of Limerick, Limerick, Ireland; 2 University Hospital Limerick, Limerick, Ireland; 3 Health Research Institute, University of Limerick, Limerick, Ireland; 4 Health Implementation Science and Technology Research Group, Health Research Institute, University of Limerick, Ireland; 5 Department of Dietetics, Nutrition and Sport, La Trobe University, Melbourne, Australia; 6 Departments of Respiratory Medicine and Physiotherapy, The Alfred, Melbourne, Australia; 7 Department of Medicine, Nursing and Health Sciences, Monash University, Melbourne, Australia; 8 Ageing Research Centre, Health Research Institute, University of Limerick, Ireland; 9 Physical Activity for Health Research Cluster, Health Research Institute, University of Limerick, Limerick, Ireland

**Keywords:** Physical activity, Randomised trial, Cystic fibrosis, fitness tracker, telehealth

## Abstract

**Background:** Physical activity (PA) and exercise are widely documented as key components in the management of cystic fibrosis (CF). In recent years there have been significant improvements in telehealth, in particular; wearable technology,  smartphone use and remote monitoring, all of which may have potential to impact on PA in adults  with CF. The objective of this pilot randomised trial is to explore the effect of wearable technology, which is remotely monitored, combined with personalised text message feedback and goal setting, on PA in adults with CF. Secondary endpoints include lung function, aerobic capacity, quality of life, body composition, wellbeing and sleep.

**Methods:** This is a pilot randomised trial which will be conducted at the University Hospital Limerick, Ireland. Participants will be randomised to the intervention or active comparator after their baseline assessment. The 12-week intervention will consist of wearable technology (Fitbit Charge 2) which is linked to an online monitoring system (Fitabase) that enables the physiotherapist to remotely monitor participant data. The CF physiotherapist will set individualised PA goals with each participant at baseline and will send text message feedback each week. The text messages will be personalised, one-way texts with positive reinforcement on step count attained by the participant. The active comparator group will receive this wearable technology which is also linked to Fitabase; however, no feedback will be provided to participants in this group. Both groups will be re-assessed at 12 weeks. After this point, both groups will continue with the Fitbit alone for a further 12 weeks. Both groups will be re-assessed at 24 weeks. A semi structured interview will assess satisfaction and acceptability of the intervention.

**Discussion:** This is a novel concept which utilises modern technology, remote monitoring and personalised feedback to investigate the effect on PA  in adults with CF.

**Trial registration:** ClinicalTrials.gov NCT03672058 (14/09/2018)

## Background

Cystic fibrosis (CF) is a life limiting, progressive disease which requires lifelong management. The prevalence of CF is 7 per 100,000 in the European Union, with Ireland reporting the highest incidence of CF in the world ( Farrell, 2008). CF is a multisystem disease, primarily affecting the respiratory system which leads to recurrent pulmonary infections with retained secretions, airway obstruction and hyperinflation ( Chmiel & Davis, 2003). There is significant burden associated with CF for the individual with CF, their family and wider society. Issues related to treatment adherence ( Sawicki *et al.*, 2009) and psychological wellbeing ( Quittner *et al.*, 2016), are frequently reported in the literature, especially in adults that are balancing family, work and education, as well as managing their chronic disease ( Boyle, 2003). Despite the complexity in the management of CF, much of the regime can be completed from home, enabling the individual with CF to integrate treatment into everyday routines with monitoring from the CF team ( Calthorpe *et al.*, 2020).

Physical activity (PA) can be described as any bodily movement that causes an increase in energy expenditure above that of resting energy expenditure including leisure-time PA, occupational PA and exercise ( Caspersen *et al.*, 1985). Exercise and PA are widely documented in consensus statements as key components in the management of CF ( Castellani *et al.*, 2018). PA has several positive benefits in this population as it has been shown to improve sputum clearance ( Dwyer *et al.*, 2009), bone mineral density ( Gupta *et al.*, 2019) and muscle strength ( Burtin *et al.*, 2013). More importantly, PA can improve aerobic capacity ( Hebestreit *et al.*, 2010) and it can slow the rate of decline in lung function ( Schneiderman *et al.*, 2014), both of which are linked to increased survival in CF ( Nixon *et al.*, 1992). As a result, the optimisation of PA among adults with CF is important, however, a Cochrane review found that there was a lack of evidence regarding strategies to promote PA in this population and consideration should be given towards telemedicine applications and health coaching ( Cox *et al.*, 2013b).

Numerous subjective and objective methods are reported in the literature for the assessment of PA in adults with CF. Step count measurement can be used to quantify and monitor PA behaviours ( Tudor-Locke & Bassett, 2004). Despite the clear benefits of PA in CF, a recent systematic review found that adults with CF fail to meet recommended PA and step count guidelines ( Shelley *et al.*, 2019). Pedometers can increase PA in other populations ( Bravata *et al.*, 2007; Hospes *et al.*, 2009; Lubans *et al.*, 2009). Therefore, interventions which aim to increase PA in adults with CF are of considerable interest.

The evolution of telehealth in CF management is significant in recent years. Previous studies have investigated the effect of telehealth on monitoring health status ( Grzincich *et al.*, 2010), detecting exacerbations ( Lechtzin *et al.*, 2017; Wood *et al.*, 2017), assessing exercise capacity ( Cox *et al.*, 2013a) and providing outpatient appointments ( Wood *et al.*, 2017). Telehealth is well accepted by adults with CF ( Cox *et al.*, 2012). While home monitoring via telehealth seems to be a progressing area of CF management, to date, no studies have evaluated the effect of telehealth on PA and health outcomes in adults with CF. Smartphones and wearable technology may assist CF physiotherapists as they can access PA data remotely ( Tagliente *et al.*, 2016). Remote monitoring and home-based PA interventions in adults with CF are advantageous for several reasons. It enables the intervention to be easily implemented, participants’ personal preferences can be considered, it is more accessible to all participants and it can involve family/friends ( Hebestreit *et al.*, 2010). As a result, these benefits may increase adherence to the intervention. Wearable technology and text message feedback has had positive health outcomes amongst other study populations ( Cadmus-Bertram *et al.*, 2015; Cook *et al.*, 2013; Moy *et al.*, 2012; Nguyen *et al.*, 2009; Vaes *et al.*, 2013), however limited research has been conducted among adults with CF to date.

In addition to telehealth, goal setting and reviewing goals regularly should be considered to support patients with chronic illness ( Coleman & Newton, 2005). In order for goals to be achieved, feedback should be provided that reveals progress in relation to the goal ( Locke & Latham, 2002). Furthermore, setting specific goals should offer a plan to break PA goals into more practical, manageable steps ( Shilts *et al.*, 2004) which should increase self-efficacy and hence promote continued regular PA levels ( Bandura, 2004). Previous literature from patient preference research and effective PA promotion approaches reports that successful strategies include specifically targeting PA ( Conn *et al.*, 2008), the use of behavioural strategies such as feedback and goal setting ( Conn *et al.*, 2008; Kosma *et al.*, 2005) ( George *et al.*, 2012) and the ability to self-monitor ( Conn *et al.*, 2008). The Irish national framework for self-management includes goal setting and action planning in chronic disease management ( Chronic Conditions Working Group, 2017). PA interventions using pedometers have been shown to be more effective at increasing PA if they include step goals ( Bravata *et al.*, 2007). However, goal setting to increase PA in adults with CF is poorly investigated.

This pilot randomised trial aims to explore the impact of wearable technology, text message personalised feedback and goal setting on PA and health outcomes in adults with CF. For the purpose of this study these specific health outcomes include lung function, aerobic capacity, body composition, quality of life, well-being and sleep. Poor sleep quality has been previously highlighted as an issue in adults with CF ( Milross *et al.*, 2002) and PA interventions can improve sleep quality in other populations ( Lan *et al.*, 2014; Yang *et al.*, 2012).

In addition, it is important to conduct a qualitative analysis to evaluate the implementation, delivery and acceptability of the intervention as this is the key to the development of future research which this pilot study aims to inform. Therefore, semi structured interviews will be conducted with the study participants and with the healthcare professionals providing the intervention. This will aim to inform future studies in this evolving research area. The current protocol serves to:

- Describe the methodology that will be implemented to evaluate a 12-week intervention consisting of wearable technology with remote monitoring, personalised text message feedback and goal setting amongst adults with CF attending University Hospital Limerick
- Describe the health outcomes in adults with CF that will be evaluated in the trial including PA levels, lung function, aerobic capacity, body composition, quality of life, wellbeing and sleep
- Describe the nested process evaluation through the conduct of semi structured interviews amongst adults with CF and healthcare practitioners regarding the implementation, delivery and acceptability of the intervention.

## Methods

### Study design

**Figure 1.**
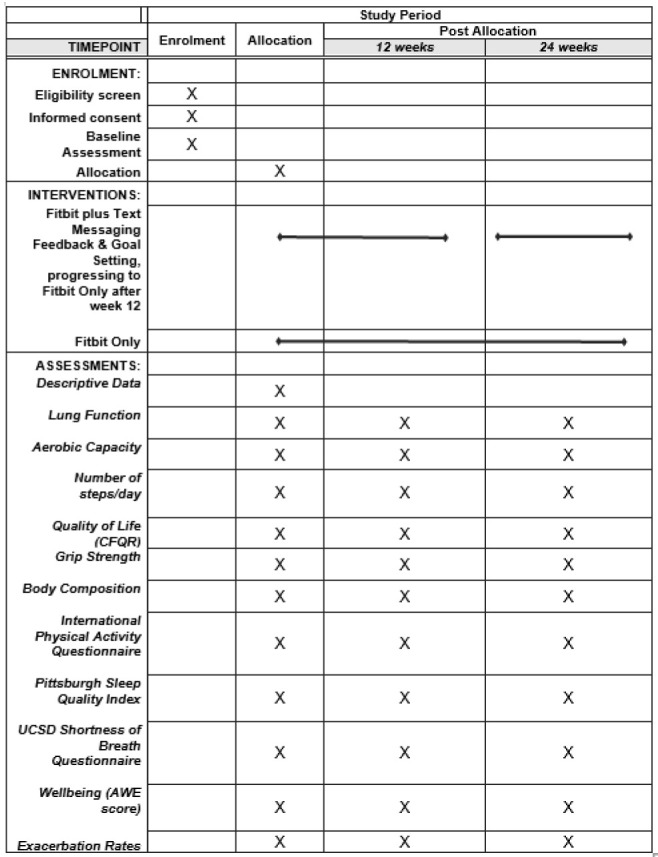
Study Schedule.

This study represents a single centre pilot randomised trial which will compare the effect of wearable technology with personalised text message feedback and goal setting to wearable technology alone in adults with CF. The CONSORT standardised reporting guidelines will be followed to ensure the standardised conduct and reporting of the research ( Schulz *et al.*, 2010). This protocol was registered on ClinicalTrials.gov ( NCT03672058) on 14 ^th^ September 2018 and prepared in accordance with the Standard Protocol Items: Recommendations for Interventional Trials (SPIRIT) guidelines (see Figure 1 and Reporting guidelines).

### Setting

The study will take place in the Adult CF Unit, University Hospital Limerick, Ireland. Baseline and follow-up assessments will take place via routine clinic outpatient appointments. Each adult with CF attends their routine clinic appointment every three months. Therefore, we chose this duration for our intervention to dovetail with routine CF care and limit the need for the adult with CF to attend on a more regular basis for testing appointments. This was decided through public and patient involvement (PPI) (completed with adults with CF) and through clinician experience. If the participant cannot attend on a particular clinic day, then they will be offered an additional appointment to suit their schedule. Due to the nature of the intervention, blinding of participants is not possible.

### Ethical approval

Ethical Approval was obtained from the University Hospital Limerick Research Ethics Committee (Approval number 054/18).

### Population and recruitment

***Recruitment strategy***. Participants deemed eligible for inclusion to the study based on the inclusion/exclusion criteria will be approached by the study gatekeeper (CF Physiotherapist – LC) and provided with an outline of the study during their routine clinic outpatient appointment. Participants will be provided with an information leaflet and will be offered an opportunity to ask questions about participation in the study. Prospective participants will then be asked to sign a consent form. Consent and mechanisms relating to data controlling and processing will be compliant with the EU General Data Protection Regulation 2016/679 and in compliance with the Data Protection Act 2018 [(Section 36(2)) (Health Research) Regulations 2018].

### Sample size

As this is the first study of its kind a sample size cannot be determined. There are 80 adults with CF attending this CF clinic. Based on inclusion/exclusion criteria at this CF centre in University Hospital Limerick, it is intended to recruit up to 50 participants.

### Inclusion criteria

- Age ≥ 18 years
- Confirmed diagnosis of CF (based on CF-causing mutations and/or a sweat chloride concentration during two tests of > 60 mmol/l)
- Clinically stable patients with CF attending University Hospital Limerick, determined by those who are not experiencing a pulmonary exacerbation. For the purpose of this study pulmonary exacerbation will be defined as acute or subacute worsening of respiratory symptoms which warrant change in treatment (i.e., new oral or intravenous antibiotics), as per previous research ( Savi *et al.*, 2015).
- Access to a smartphone/tablet to access and ability to upload to Fitbit Application
- Capacity and willingness to give explicit informed consent

### Exclusion criteria

- FEV _1_ < 25%. Individuals with FEV _1_ <25% would typically require supplemental oxygen for exercise and at this point may require transplant assessment. Furthermore, they are more likely to experience exacerbations and would not be suitable for a study over 24 weeks. This is a lower cutoff FEV _1_ than most studies would include ( Hebestreit *et al.*, 2010; Klijn *et al.*, 2004; Kriemler *et al.*, 2013).
- Patients on the waiting list for lung transplantation and those who have undergone lung transplantation.
- Patients with an exacerbation in the four weeks prior to the study. Patients can undergo testing once they are finished their antibiotics and deemed clinically stable by the Respiratory Consultant (BC).
- Patients dependent on supplemental oxygen for exercise.
- Adults with CF who are pregnant
- Patients with any cardiac, neurological or musculoskeletal impairment that may impact on their ability to participate in the PA intervention will not be eligible to take part in this study
- Participation in another clinical trial up to 4 weeks prior to the first baseline visit

### Randomisation

Should participants explicitly consent to participate in the study, they will undergo baseline testing. To minimise the possibility of selection bias, a researcher independent of the recruitment process (MC) will complete the first random allocation using a sealed opaque envelope. Following this a minimisation randomisation procedure will be completed based on lung function, where FEV _1_ of >80% predicted lung function will be classified as having mild lung disease. While those with an FEV _1_ of 50–79% predicted lung function will be classified as having moderate lung disease, 30–49% as severe lung disease and <30% indicating very severe lung disease ( Ranu *et al.*, 2011). Allocation will be revealed after recruitment and baseline assessments have occurred. Participants will then either receive the intervention (Fitbit with text messaging feedback and goal setting) or the active comparator (Fitbit only).

## Experimental and active comparator intervention

### Intervention

The intervention consists of wearable technology, text message feedback and goal setting.

Wearable technology:

If the participant is allocated to the intervention, they will be provided with wearable technology (Fitbit Charge 2), educated on how to use it, and this will also be linked to an online monitoring system (Fitabase). Participants will be encouraged to enable Bluetooth and upload data regularly. Fitabase, the online monitoring system enables the physiotherapists to access step count data remotely. When a participant enables Bluetooth and syncs their Fitbit through the Fitbit app, this automatically updates participant data on the Fitabase website. Engagement with the technology will be monitored through systematic review of online data. Participants in both groups will receive a “reminder” message on his or her mobile phone if their data has not been synced via the Fitbit App in the previous seven days. This ensures that data is continuously collected over the full study duration. The data collected through the Fitbit Charge 2 and recorded on the Fitabase system are step count, activity minutes and sleep. However, for the purpose of this study, the researchers are only investigating step count alone. Any further research would require the Fitbit to be assessed under each of these conditions to ensure validity and reliability. This is outside the scope of this research.

Goal setting:

The physiotherapist will discuss the participant’s PA levels (as measured at baseline by an accelerometer) and individual patient centred PA goals will be set with each participant. These will be discussed with their physiotherapist to ensure they are specific, measurable, achievable, realistic and timed (SMART). They will be encouraged to write a minimum of three goals. Participants will be asked to set a step count target for week four, eight and 12 and consider ways to achieve these goals, both of which will be discussed with the physiotherapist during the baseline appointment. Goals will be individualised to the participant taking into account their preferences. Participants may also set goals related to health outcomes being assessed – for example, a participant may have a goal to improve lung function, aerobic capacity, sleep etc. during the study period. The text message feedback will refer to step goals only. The participant will be given a copy of their goals.

Text message feedback:

Every week participants will be sent a one-way personalised text message by their CF physiotherapist for 12 weeks overall. The text messages in this study will be personalised, one-way texts with positive reinforcement on step count attained by the participant.

A metanalysis found personalisation strategies, such as using a participant name has been proven to increase intervention efficacy ( Head *et al.*, 2013) and tailoring messages is a key ingredient in text messaging interventions ( Noar *et al.*, 2007). Furthermore, one way communication is as effective as two-way dialogue ( Head *et al.*, 2013). Therefore, this study investigated one-way messages only.

This individualised message will be sent to each participant once a week, at a day and time chosen by the participant at the start of the intervention. The authors will follow a standardised approach. The message will address the participant by name and will have positive feedback in the form of encouragement and praise messages ( Adams *et al.*, 2013) in relation to their step count attained in the previous week. It will then focus on a PA related goal set by the subject and will sign off with a further positive comment. Research has indicated that it is crucial to reinforce improvements to develop new behaviour or to strengthen a habit ( Bandura, 2004; Glanz *et al.*, 2008; Hovell *et al.*, 2009). Each time a participant meets his/her goal they will receive positive feedback. Participants who do not meet the goal will be provided with a positive comment in relation to their step count achieved to date and provided their next step goal. This is to avoid negative messages that could be discouraging ( Adams *et al.*, 2013). If the participant does not achieve their target step count goal then the physiotherapist will set a target increase of 10% from the mean step count achieved in the previous week.

A sample text message is as follows:

Hi Paul, Well done on achieving an average daily step count of 7,600 steps this week. Next week aim to hit your goal of 8,000 steps. Keep up the good work! 

### Comparison

If the participant is allocated to the active comparator, they will be provided with a Fitbit Charge 2 and educated on how to use it and this will also be linked to “Fitabase” for data collection purposes. However, no feedback will be provided to the participants on their PA levels throughout the study period.

### Follow Up

At week 12 both groups will have outcome measures re-assessed. Both groups will continue with the Fitbit Charge 2 only for the following 12 weeks. At the end of the 24 weeks participants will have all outcome measures repeated.

Subsequently, a qualitative assessment will be conducted through semi structured individual interviews to determine participants’ satisfaction and feedback on the intervention and their suggestions going forward. The interview guide is available as extended data to this manuscript ( Curran, 2020). These interviews will be completed by a CF Physiotherapist involved in the study (LK) using a digital voice recorder and will be transcribed verbatim. The transcribed texts will be coded independently by two investigators who will develop code books. These will be compared and agreed upon. Data will be analysed using NVIVO V.11 Plus (QSR International Pty Ltd) and using the six steps for thematic analysis, in order to highlight the central themes to this study ( Braun & Clarke, 2006).

### Exacerbations

If the participant has an exacerbation of CF during the study period, their involvement in the study will be paused, and re-started 1 month later.

### Baseline Assessment of step count

The ActivPAL accelerometer (PAL technologies) will be used to assess step count at baseline. This will be used to inform goal setting only. This forms part of another study which assessed PA behaviour in adults with CF. Participants will wear the accelerometer for seven days. Based on PPI it was reported by the participants that they did not want to wear an ActivPAL other than at baseline as it is a thigh worn device which is not ‘fashionable’. Similar concerns have been cited previously in the research ( Dias *et al.*, 2012). Data will be included if at least four full days (at least three weekdays and one weekend day) of measurements with a minimum of 10 hours (h) for the weekdays and 10 h for the weekends are measured ( Freedson *et al.*, 2005).

### Usual Care

Usual care will be provided to all participants in both groups. This would typically include PA and exercise advice as per Australian and New Zealand Cystic Fibrosis guidelines. People with CF are advised to exercise for at least 30 minutes, five days a week. They are advised to include a combination of aerobic and resistance training. It is personalised depending on patient preference, as per recommendations ( Button *et al.*, 2016).

### Instrumentation/outcome measures

A range of outcome measures will be employed to identify the potential impact of this study on health outcomes in adults with CF. Each of these outcomes will be assessed at baseline, at 12 weeks and 24 weeks.

## Primary

### Fitbit step count data

The Fitbit Charge 2 (Fitbit Inc.) will be used to record step count during this study with data uploaded to Fitabase (Small Steps Labs LLC). Prior to this intervention, the validity and reliability of the Fitbit Charge 2 to measure step count in CF was assessed by the same research group. Twenty-one participants were recruited from an adult CF Centre for a single session of testing. Participants walked for five minutes at five pre-determined speeds in a controlled testing environment (2, 2.5, 3, 3.5 and 4 miles per hour on a treadmill) and at three self-selected speeds on a corridor (slow, medium and fast). The Fitbit Charge 2 was compared to visual observation. It was found that the Fitbit Charge 2 ranged from weak to very strong correlations when compared to visual observation (0.34–0.84). The Fitbit Charge 2 underestimated step count by 2.8%–9.2%. This is within acceptable limits of variability based on previous research ( Schneider *et al.*, 2004). Therefore, the Fitbit Charge 2 is a valid and reliable measure to assess step count in adults with CF ( Curran *et al.*, 2020).

## Secondary

### Cardiopulmonary exercise testing

The reference standard exercise test is an incremental cardiopulmonary exercise test (CPET), utilising a ramp protocol. The ramp protocol ensures that work rate is progressively and linearly incremented until maximal effort is achieved ( Herdy *et al.*, 2016). All standard CPET outcomes will be considered including VO _2_ max, duration, load, pulmonary ventilation (VE), Respiratory Exchange Ratio (RER), ventilatory equivalents for oxygen (VE/VO _2_) and for carbon dioxide (VE/VCO _2_). This will be conducted using the Medisoft Ergocard Professional CPET equipment and analysed by ExpAir, the Medisoft software. Maximal exercise testing is an independent predictor of mortality in CF ( Nixon *et al.*, 1992). Breath-by-breath ventilatory gas analysis allows the accurate measurement of maximal oxygen uptake (VO _2_max) – the gold standard measure of exercise capacity ( Urquhart, 2011). Supramaximal verification be conducted to ensure a maximal effort during the CPET. After an incremental CPET, participants will be provided with a ten-minute break. They will then be asked to complete a supramaximal exercise test ( Causer *et al.*, 2018; Saynor *et al.*, 2013) whereby participants will exercise at 110% of their power output (as determined by CPET test previously). This test has been conducted amongst adults with CF previously and has been deemed safe and ensures validity of the results which are obtained in a CPET test.

### Spirometry

Spirometry will be performed according to American Thoracic Society (ATS) standard techniques ( Miller *et al.*, 2005) using the Carefusion Microlab spirometer. Values will be expressed as a percentage of the predicted value for height, sex and age for adults ( Hankinson *et al.*, 1999). Forced expiratory volume in one second (FEV _1_) will be used to classify the severity of CF lung disease for each participant. It will also be used to determine the effect the intervention may have on pulmonary function. Other pulmonary function measures such as % forced vital capacity (FVC) and forced expiratory flow (FEF _25–75_) will also be considered.

### International Physical Activity Questionnaire (IPAQ)

The IPAQ is a self-reported measure of PA and relies on user recall over the previous seven days. This tool was developed to assess PA levels using a questionnaire, has very good repeatability and is as reliable as other measures for self-reported PA ( Craig *et al.*, 2003). This nine-item questionnaire relies on participant recall and records PA at four levels: vigorous (e.g. aerobics), moderate (e.g. leisure cycling), walking and sitting.

### Hand dynamometry

Grip strength will be recorded as a measure of general musculoskeletal strength, independent of lower limb strength. Previous research has shown that grip strength is correlated to lung function and VO2 peak ( Wells *et al.*, 2014). This will be conducted using a Jamar Hydraulic Hand Dynamometer. Test – retest reliability has been proven in respiratory patients ( Dowman *et al.*, 2016). The participant will be asked to stand and hold the dynamometer in by their side, with their elbow at 90 degrees and forearm in neutral. They will be instructed to squeeze the device as hard as possible. They will be provided with a 30 second rest period between each trial. The greater of two trials from each hand will be used and added together to give overall handgrip strength. This will be measured in kilograms ( Martínez-García *et al.*, 2020).

### Bioelectrical Impedance Analysis (BIA)

BIA is an easily available, quick method to assess body composition. A low voltage current will be passed through the body, whereby impedance (tissue resistance and reactance) is measured. This will be conducted using the Seca 515 Medical Body Composition Analyser. BIA will be performed with the participant standing barefoot on the instrument platform as per manufacturers guidelines (Seca, Birmingham, United Kingdom). The device has an integrated scale and uses four pairs of electrodes of stainless steel that are positioned at each hand and foot, through which the current enters the limbs.

### CF Quality of Life Questionnaire Revised (CFQR)

The CFQR questionnaire is a fully validated disease specific measure consisting of 52 items across nine domains of functioning which have been identified by, and are of importance to, adolescents and adults with CF. This questionnaire is valid, sensitive and has strong test-retest reliability ( Gee *et al.*, 2000). The minimum clinically important difference for the CFQR – respiratory score is 4 points ( Quittner *et al.*, 2009).

### Pittburgh Sleep Quality Index (PSQI)

The PSQI is a self-rated questionnaire used to measure the quality and patterns of sleep in adults. It differentiates “poor” from “good” sleep quality by measuring seven areas (components): subjective sleep quality, sleep latency, sleep duration, habitual sleep efficiency, sleep disturbances, use of sleeping medications, and daytime dysfunction over the last month. The PSQI is effective in assessing sleep quality in CF ( Milross *et al.*, 2002).

### The University of California San Diego (UCSD) Shortness of Breath Questionnaire

This questionnaire assesses dyspnoea associated with activities of daily living (ADLs). There are 24 items on this questionnaire. Each item is assessed on a 6-point scale (0 = “not at all” to 5 = “maximal or unable to do because of breathlessness”). Scores range from 0 to 120 with higher scores indicating activities of daily living are extremely limited by shortness of breath. It has been validated to assess dyspnoea over time in respiratory patients ( Swigris *et al.*, 2012)

### Awescore

This questionnaire assesses state of wellness to assist in providing best health care ( Button *et al.*, 2014). There are ten questions, which are scored from 0–10. 10 reflects most well state of being possible while zero reflects least well state. Scores range from 0–100 with higher scores indicating good state of wellness. This is a reliable tool for measurement of multidimensional wellness in adults with CF that is appealing to patients ( Button *et al.*, 2014).

### Data collection & management

Outcome assessment will be conducted by qualified physiotherapists in the Adult CF Unit. Assessors cannot be blinded as the intervention involves the CF physiotherapists for the delivery of weekly text messages, who will also be assisting with repeating objective outcome measures. All self-report measures will be completed by the participants independently, however a research assistant (RA) (AJ) will be available if there are any queries or concerns. These subjective assessments will be analysed by the RA who is blinded to group allocation.

Each participant in the study will be assigned a numerical identifier code. Aggregate data will be anonymised. Paper copies of all measures will be identifiable only by the identifier code. All data will be stored in locked filing cabinets in locked offices in the Adult CF Unit at University Hospital Limerick. All identifying paper data (e.g. signed consent forms and forms listing participant codes) will be stored in a separate locked filing cabinet to all other anonymised data and will be accessible only to the research team. All interviews will be coded and entered to a study data file. These computer files will be stored on the hard drive of a password protected desktop computer by the study lead investigator (MC). All audio and electronic data will be stored on encrypted hard drives.

### Data analysis

Step count data will be collected over seven days and an average weekly step count will be obtained. It is anticipated that this step count data will be analysed to assess for a novelty effect in the first two weeks, at week six and 12 during the intervention and at week 18 and Week 24 for the follow up. Appropriate descriptive statistics will be used to describe the baseline characteristics of study participants. These will include proportions, percentages, ranges, means and standard deviations and medians and interquartile ranges (where data are not normally distributed). Data will be assessed for normality. Parametric tests will be used to compare differences across groups where data are normally distributed, and the non-parametric equivalent will be applied in cases where data are not normally distributed. A p-value <0.05 will be considered significant. Primary analyses will be performed according to intention-to-treat (ITT) principles with all participants who were originally allocated by randomisation and those who dropped out from the study. In case of missing values, a multiple imputation method, inverse probability weighing or mixed models will be used to handle the missing data. Data will be presented as means and SDs for data that is normalised and medians (IQR) for data that is skewed. A repeated measures ANOVA, controlling for baseline values and other potential confounders will be conducted. Bonferroni correction will be applied for multiple testing.

### Dissemination of information

The results of this study will be disseminated via peer-reviewed publications and conference presentations.

### Study status

Recruitment began in January 2019. It is anticipated this will be completed by June 2020.

## Discussion

This is the first study to assess the effect of wearable technology with personalised text message feedback and goal setting on PA and key health outcomes in adults with CF. Previous research has investigated the feasibility and acceptability of a telehealth intervention in adults with CF with positive results reported for such an intervention ( Cox *et al.*, 2015). The use of wearable technology with remote monitoring is a novel concept in CF.

Regular PA is a well-accepted and valued part of CF care ( Dwyer *et al.*, 2011; Rand & Prasad, 2012). The primary aim of this research is to increase PA in adults with CF. Adherence to any PA intervention is a vital component of any study, particularly in chronic disease. The use of pedometers to provide feedback on PA levels can increase awareness of daily PA, provide motivation and visual feedback ( Lauzon *et al.*, 2008). Pedometers can increase PA in an adult population ( Bravata *et al.*, 2007), in a young population ( Lubans *et al.*, 2009) and in chronic obstructive pulmonary disease ( Hospes *et al.*, 2009). To date, there is limited research conducted assessing wearable technology such as Fitbits in this population.

Previous literature has demonstrated that a partially supervised programme in CF can improve health outcomes and is easily implemented ( Hebestreit *et al.*, 2010). Other partially supervised interventions have investigated the effect of providing personalised text message feedback on PA levels which has enhanced adherence to home based programmes in other populations ( Blaauwbroek *et al.*, 2009; Strath *et al.*, 2011). Furthermore, a systematic review found that having a step goal was the key predictor of increasing PA. Studies which did not include a step goal as part of their intervention showed no significant improvement in PA in comparison to those that set step goals ( Bravata *et al.*, 2007).

The secondary aims of this research is to consider the effect of this intervention on other important endpoints such as lung function, aerobic capacity, quality of life, wellbeing and sleep. The broad range of outcomes assessed in this study will facilitate a comprehensive review on the impact of this research on participants with CF, both subjectively and objectively. A strength of this study is the use of the Fitbit Charge 2 which will allow participants to update their PA levels through the Fitbit App which will enable the assessors to review data remotely and this will also provide feedback on compliance to the intervention.

There is no true control group in this study for a couple of reasons. Firstly, the primary aim of this study was to determine if a partially supervised programme would be effective in improving PA and health, and it is unknown if any of these key outcomes could be improved with the provision of a Fitbit alone. Therefore, it was deemed more appropriate to compare the intervention with the Fitbit alone rather than a true control group. Secondly, based on clinical experience and PPI, both the CF Physiotherapists and adults with CF felt that this would be a limitation to recruitment and retention if only one group received the Fitbit.

Gene modifiers and potentiators may be a confounder in this study. This will be monitored throughout the study duration and will be controlled for during statistical analysis, if necessary. The research team will also monitor exacerbation rates (participants requiring oral or IV antibiotics during the study period), hospital admission rate and colonisation status.

### Limitations

It is not possible to blind participants in PA intervention research. Due to lack of resources it is not possible to blind the CF Physiotherapists who will be assisting with text messaging feedback and repeating objective outcome measures, however self-report measures will be analysed by a research assistant blinded to group allocation. There may be a selection bias towards more physically active adults with CF as those who are more active may be more likely to partake in this study.

The results of this study may provide valuable insights into the development of a larger definitive trial exploring the use of wearable technology for optimising PA and health outcomes in adults with CF.

### Ethics approval and consent to participate

The study received ethical approval from the University Hospital Limerick Research Ethics Committee (Ref: 054/18). Written informed consent will be obtained from all study participants.

## Data availability

### Underlying data

No data are associated with this article

### Extended data

Open Science Framework: Steps Ahead: optimising physical activity and health in people with cystic fibrosis. Study protocol for a pilot randomised trial https://doi.org/10.17605/OSF.IO/DEGHT ( Curran, 2020)

This project contains the following extended data:

- Participant Consent Form
- Participant Information leaflet
- Interview Guide

### Reporting guidelines

Spirit Checklist for Steps Ahead: optimising physical activity and health in people with cystic fibrosis. Study protocol for a pilot randomised trial https://doi.org/10.17605/OSF.IO/DEGHT ( Curran, 2020)

Data are available under the terms of the Creative Commons Zero “No rights reserved” data waiver (CC0 1.0 Public domain dedication).
