# Supplementary material for: Steps Ahead: optimising physical activity in adults with cystic fibrosis: Study Protocol for a pilot randomised trial using wearable technology, goal setting and text message feedback
Source: HRB Open Res. 2020 Nov 16;3:21. Originally published 2020 May 5. [Version 3] doi: 10.12688/hrbopenres.13025.3 (PMC7689601; doi:10.12688/hrbopenres.13025.3)
Supplement: Peer review history

# Peer review: Reviewer response for version 3

**DOI:** 10.21956/hrbopenres.14322.r28376

**Published:** 2020-11-25

**Authors:** Kenneth Wu

**Affiliations:** 1 Toronto Adult Cystic Fibrosis Centre, St. Michael's Hospital, Unity Health Toronto, Toronto, ON, Canada; 2 Rehabilitation Sciences Institute, Faculty of Medicine, University of Toronto, Toronto, ON, Canada; 3 Department of Physical Therapy, Faculty of Medicine, University of Toronto, Toronto, ON, Canada

This title is better able to reflect the study objectives.

Is the study design appropriate for the research question?

Partly

Is the rationale for, and objectives of, the study clearly described?

Yes

Are sufficient details of the methods provided to allow replication by others?

No

Are the datasets clearly presented in a useable and accessible format?

Not applicable

Reviewer Expertise:

Limb muscles in adults with cystic fibrosis.

I confirm that I have read this submission and believe that I have an appropriate level of expertise to confirm that it is of an acceptable scientific standard.

# Peer review: Reviewer response for version 2

**DOI:** 10.21956/hrbopenres.14198.r28128

**Published:** 2020-11-12

**Authors:** Kenneth Wu

**Affiliations:** 1 Toronto Adult Cystic Fibrosis Centre, St. Michael's Hospital, Unity Health Toronto, Toronto, ON, Canada; 2 Rehabilitation Sciences Institute, Faculty of Medicine, University of Toronto, Toronto, ON, Canada; 3 Department of Physical Therapy, Faculty of Medicine, University of Toronto, Toronto, ON, Canada

This is an improved version of the document. I still have some concerns on how the study design would be able to achieve what the objective of this study set out. 

 My understanding is the experimental group (Exp) receives wearable technology (WT) + goal setting (GT) + text message feedback (TF), while the "active comparators" (Control) will only be given WT, i.e.

  Exp: WT + GT + TF

  Control: WT

 I'm curious how this study design would be able to tell if wearable technology can change the physical activity level, if both groups receive WT intervention. Perhaps you did mean wearable technology = Fitbit Charge 2 + GT + TF, while the control group only uses Fitbit and thus not considered wearable technology. Or you need to change the wording of the objective (and the title), by replacing "wearable technology" by goal setting and feedback.

 Also, can the subjects be able to see their step count on the Fitbit Charge 2, if so, do you think the goal setting and text message are "external" feedback that would be more helpful than what the patients can do for themselves.

Is the study design appropriate for the research question?

Partly

Is the rationale for, and objectives of, the study clearly described?

Yes

Are sufficient details of the methods provided to allow replication by others?

No

Are the datasets clearly presented in a useable and accessible format?

Not applicable

Reviewer Expertise:

Limb muscles in adults with cystic fibrosis.

I confirm that I have read this submission and believe that I have an appropriate level of expertise to confirm that it is of an acceptable scientific standard, however I have significant reservations, as outlined above.

# Response

**Published:** 2020-11-13

**Authors:** Maire Curran

**Affiliations:** University of Limerick, Ireland

Dear Dr Wu,

 Thank you for your feedback.

 The intervention group will receive the wearable technology, goal setting and text message feedback. Therefore, we have added “goal setting and text message feedback” to the title:

**Steps Ahead: optimising physical activity in adults with cystic fibrosis: Study Protocol for a pilot randomised trial using wearable technology, goal setting and text message feedback.**

 These objectives have already been stated throughout the introduction and methods.

 We do not feel that we should remove the words “wearable technology” as it is through the use of wearable technology that physiotherapy staff will see PA data remotely and send text message feedback on same and it is an important component of our study. Furthermore, the wearable technology will allow the participants to monitor their daily PA, as well as hopefully encourage further PA in order to attain their goals.  

 The objective of this study is to determine if the intervention (wearable technology, goal setting and text message feedback) will increase step count more than wearable technology alone. Subjects in both groups will be able to see their step count on the Fitbit Charge 2. We think that goal setting and text message feedback will positively impact on PA levels rather than just providing participants with the Fitbit alone. We feel this external feedback may be more beneficial than what the patient can do themselves. The provision of a Fitbit to the active comparator group should enable us to determine if this is the case.

# Peer review: Reviewer response for version 2

**DOI:** 10.21956/hrbopenres.14198.r28127

**Published:** 2020-11-05

**Authors:** Sarah Rand

**Affiliations:** 1 UCL Great Ormond Street Institute of Child Health, University College London, London, UK

Thank for asking me to review this updated version. The authors have obviously taken all comments on board and have made many useful revisions and additions to the article based on the feedback provided. The article in my opinion is now a much clearer and more comprehensive outline of the protocol. 

 I have no further comments or suggestions to make at this stage.

Is the study design appropriate for the research question?

Partly

Is the rationale for, and objectives of, the study clearly described?

Partly

Are sufficient details of the methods provided to allow replication by others?

No

Are the datasets clearly presented in a useable and accessible format?

Not applicable

Reviewer Expertise:

Cystic Fibrosis, Physical activity, exercise, health technology.

I confirm that I have read this submission and believe that I have an appropriate level of expertise to confirm that it is of an acceptable scientific standard.

# Peer review: Reviewer response for version 1

**DOI:** 10.21956/hrbopenres.14120.r27734

**Published:** 2020-08-24

**Authors:** Kenneth Wu

**Affiliations:** 1 Toronto Adult Cystic Fibrosis Centre, St. Michael's Hospital, Unity Health Toronto, Toronto, ON, Canada; 2 Rehabilitation Sciences Institute, Faculty of Medicine, University of Toronto, Toronto, ON, Canada; 3 Department of Physical Therapy, Faculty of Medicine, University of Toronto, Toronto, ON, Canada

This is an interesting study protocol. The title sounds catching. Nevertheless, I wonder the wording should be more specific, e.g. the word "health" is rather vague. The primary outcomes of this study are exercise capacity (with CPET) and lung function (spirometry), perhaps it can be more specific. Also, the study participants are all adults. The word "adults" should be used instead of "people", as the study design, hence the implications, may be different if it was with children.

 For the concept of physical activity, the authors talked about exercise and physical activity as two different entities in the Introduction, then the focus switched to only physical activity. I wonder how the authors conceptualize physical activity in this study. Does physical activity include exercise for this study? Many studies separated physical activity to various intensities - mild, moderate, and vigorous intensities (i.e. including exercise), e.g. Trooster *et al.* (2009) ^1^.

**Study Design**

 In terms of intervention, I am curious how the physiotherapist re-assess participant's goals while a one-way text message was used. Should goal-setting involve a discussion between the patient and health-care providers? Or was it more a physical activity prescription?

 I don't think the study design can achieve the study objectives when the study participants in the control group also wear the Fitbit (while the study objectives were to explore the impact of a fitness tracker, text message personalised feedback and goal setting on physical activity and health outcomes).

 Since the sample size was not calculated based on previous studies, it would be helpful to provide information on the size of the Adult CF clinic.

 It is unclear in the Methods how study participants were recruited. Were they approached when they were going for an outpatient CF appointment? Or was it done over the phone or via Email? Also, related to this, was study conducted using convenience sampling?

 In terms of exclusion criteria, should patients having comorbidities that might affect their participation in physical activity (e.g. people with cardiac, neuromuscular issues, if they had injuries to their ligaments/tendons etc, or mental health issue). Also, what if the study participants do lots of "physical activity" or exercise already, before they were enrolled in the study, would they be expected to have any effects from the intervention?

 It is unclear why the group that also wore the fitness tracker (only) and why having people wearing the fitness tracker for 12 more weeks. Were the extra 12 weeks function like a control period? If so, would there be any carry-over effect in your experimental group if that's the case?

 For the handgrip strength assessment, I am not sure if it really indicates "physical function." In the CF literature, handgrip strength was used as a surrogate measure for general muscle strength (Martinez-Garcia *et al*., 2020 ^2^; Rietschel *et al*., 2008 ^3^; Ward *et al*., 2013 ^4^). Physical function is a broader concept than muscle strength. I am curious why a study in ILD should be cited here.

 Since your outcome measures were mostly self-report, or else they are easy to perform (and to learn), and there were many people working on this study, would it enhance the quality of the study by blinding the assessor (i.e. not using the same physiotherapist providing the feedback to the study participants).

**Statistical Analysis**

 There are 10 outcome measures in this study. A *p*-value of p = 0.05/10 = 0.005 should be used to determine statistical significance. Perhaps the authors should consider limiting the number of outcome measures. For example, can IPAQ be eliminated since the fitness tracker was keeping track of the same/similar information?

 Even if only the primary outcomes are considered, the *p*-value of *p* = 0.05/2 = 0.025 should be used. 

**Minor Correction Recommendation**

 In the Introduction and Abstract section, "pilot randomised trial" was written in this order, but in the Study Design section and perhaps some other places, it was put as "randomised pilot trial". To me, the former order seems correct.

Is the study design appropriate for the research question?

Partly

Is the rationale for, and objectives of, the study clearly described?

Yes

Are sufficient details of the methods provided to allow replication by others?

No

Are the datasets clearly presented in a useable and accessible format?

Not applicable

Reviewer Expertise:

Limb muscles in adults with cystic fibrosis.

I confirm that I have read this submission and believe that I have an appropriate level of expertise to confirm that it is of an acceptable scientific standard, however I have significant reservations, as outlined above.

## References

[1] : Skeletal muscle weakness, exercise tolerance and physical activity in adults with cystic fibrosis. *Eur Respir J*.2009;33(1) : 10.1183/09031936.00091607 99-106 10.1183/09031936.00091607 18715878

[2] : Influence of sex gap on muscle strength and functional mobility in patients with cystic fibrosis. *Appl Physiol Nutr Metab*.2020;45(4) : 10.1139/apnm-2019-0484 387-392 10.1139/apnm-2019-0484 31526325

[3] : Whole body vibration: a new therapeutic approach to improve muscle function in cystic fibrosis?. *Int J Rehabil Res*.2008;31(3) : 10.1097/MRR.0b013e3282fb783d 253-6 10.1097/MRR.0b013e3282fb783d 18708849

[4] : Physical activity levels of patients with cystic fibrosis hospitalised with an acute respiratory exacerbation. *Respir Med*.2013;107(7) : 10.1016/j.rmed.2013.03.002 1014-20 10.1016/j.rmed.2013.03.002 23601566

# Response

**Published:** 2020-09-30

**Authors:** Maire Curran

**Affiliations:** University of Limerick, Ireland

Dear Dr Wu,

**Re: Steps Ahead: optimising physical activity and health in people with cystic fibrosis: Study Protocol for a pilot randomised trial**

**This is an interesting study protocol. The title sounds catching. Nevertheless, I wonder the wording should be more specific, e.g. the word "health" is rather vague. The primary outcomes of this study are exercise capacity (with CPET) and lung function (spirometry), perhaps it can be more specific. Also, the study participants are all adults. The word "adults" should be used instead of "people", as the study design, hence the implications, may be different if it was with children.**

We sincerely thank you for your detailed and critical analysis of our article. Please find our responses below to your feedback (in bold). All changes have been made and are illustrated through italics and will be submitted in the second version of this article.

We agree the title should be more specific and given reviewers feedback we have changed the title to *“Steps Ahead: optimising physical activity in adults with cystic fibrosis: Study Protocol for a pilot randomised trial using wearable technology”.*

**For the concept of physical activity, the authors talked about exercise and physical activity as two different entities in the Introduction, then the focus switched to only physical activity. I wonder how the authors conceptualize physical activity in this study. Does physical activity include exercise for this study? Many studies separated physical activity to various intensities - mild, moderate, and vigorous intensities (i.e. including exercise), e.g. Trooster *et al.* (2009)** **^1^** **.**

Thank you for this feedback. We have clarified PA in the introduction. This study is focusing on step count only as a proxy measure for PA.

*“PA can be described as any bodily movement that causes an increase in energy expenditure above that of resting energy expenditure* *including leisure-time PA, occupational PA and exercise* *(Caspersen et al., 1985)* *. Exercise and physical activity (PA) are widely documented in consensus statements as key components in the management of CF ( *Castellani et al., 2018 *). PA has several positive benefits in this population as it has been shown to improve sputum clearance (*Dwyer et al., 2009 *), bone mineral density* *(Gupta et al., 2019)* *and muscle strength (*Burtin et al., 2013 *). More importantly, PA can improve aerobic capacity* *(Hebestreit et al., 2010)* *and it can slow the rate of decline in lung function* *(Schneiderman et al., 2014)* *, both of which are linked to increased survival in CF* *(Nixon et al., 1992)* *. As a result, the optimisation of exercise and PA among PWCF is important, however, a Cochrane review found that there was a lack of evidence regarding strategies to promote PA in this population and consideration should be given towards telemedicine applications and health coaching (* *Cox et al., 2013b* *).*

*Numerous subjective and objective methods are reported in the literature for the assessment of PA in adults with CF. Step count measurement can be used to quantify and monitor PA behaviours* *(Tudor-Locke and Bassett, 2004)* *. Despite the clear benefits of PA in CF, a recent systematic review found that adults with CF fail to meet recommended PA and step count guidelines* *(Shelley et al., 2019)* *. Therefore, interventions which aim to increase PA in this population are of considerable interest.*

**Study Design**

**In terms of intervention, I am curious how the physiotherapist re-assess participant's goals while a one-way text message was used. Should goal-setting involve a discussion between the patient and health-care providers? Or was it more a physical activity prescription?**

Thank you for this feedback. They following text has been added to the intervention section to ensure clarity around text messaging and goal setting:

*“The text messages in this study will be personalised, one-way texts with positive reinforcement on step count attained by the participant.* *A metanalysis found personalisation strategies such as using a participant name has been proven to increase intervention efficacy* *(Head et al., 2013)* *and tailoring messages is a key ingredient in text messaging interventions* *(Noar et al., 2007)* *. Furthermore, one way communication is as effective as two-way dialogue* *(Head et al., 2013)* *. Therefore, this study investigated one-way messages only.*

*An individualised message will be sent to each participant once a week, at a day and time chosen by the participant at the start of the intervention. The authors will follow a standardised approach. The message will address the participant by name and will have positive feedback* *in the form of encouragement and praise messages* *(Adams et al., 2013)* *in relation to their step count attained in the previous week. It will then focus on a physical activity related goal set by the subject and will sign off with a further positive comment.  Research has indicated that it is crucial to reinforce improvements to develop new behaviour or to strengthen a habit* *(Hovell et al., 2009, Glanz et al., 2008, Bandura, 2004)* *.* *Each time a participant meets his/her goal they will receive positive feedback. Participants who do not meet the goal will be provided with a positive comment in relation to their step count achieved to date and provided their next step goal. This is to avoid negative messages that could be discouraging* *(Adams et al., 2013)* *.”*

**I don't think the study design can achieve the study objectives when the study participants in the control group also wear the Fitbit (while the study objectives were to explore the impact of a fitness tracker, text message personalised feedback and goal setting on physical activity and health outcomes).**

Thank you for this comment. However, we feel that the study design can achieve the study objectives. The control group will wear the Fitbit also, however we are keen to investigate if using the wearable technology with text message feedback combined with goals will impact on physical activity and health outcomes. We felt it would be important to consider whether the Fitbit alone could achieve increases in PA.

The control group is not ‘usual care’ so essentially, we are assessing the use of text messaging feedback and the Fitbit is to inform this process.

Furthermore, based on PPI, we found that adults with CF reported they would be less likely to comply if they didn’t also receive a Fitbit.

We considered other study designs such as a crossover study. However, we felt this was too complex for a small pilot study and taking into account overall patient burden.

**Since the sample size was not calculated based on previous studies, it would be helpful to provide information on the size of the Adult CF clinic.**

Thank you for this comment. We have provided further information under sample size.  

*“There are 80 adults with CF attending this CF clinic. Based on inclusion/exclusion criteria at this CF centre in University Hospital Limerick, it is intended to recruit up to 50 participants.”*

**It is unclear in the Methods how study participants were recruited. Were they approached when they were going for an outpatient CF appointment? Or was it done over the phone or via Email? Also, related to this, was study conducted using convenience sampling?**

Participants were approached during their outpatient CF appointment which they attend every three months. Convenience sampling was not used as all people with CF attending this CF clinic, who met the inclusion/exclusion criteria were asked if they would like to participate in this study.

***“*** *Participants deemed eligible for inclusion to the study based on the inclusion/exclusion criteria will be approached by the study gatekeeper (CF Physiotherapist – LC) and provided with an outline of the study during their routine clinic outpatient appointment. Participants will be provided with an information leaflet and will be offered an opportunity to ask questions about participation in the study. Prospective participants will then be asked to sign a consent form.”*

**In terms of exclusion criteria, should patients having comorbidities that might affect their participation in physical activity (e.g. people with cardiac, neuromuscular issues, if they had injuries to their ligaments/tendons etc, or mental health issue).**

Thank you for this suggestion. We have added the following to the exclusion criteria:

*“Patients with any cardiac, neurological or musculoskeletal impairment that may impact on their ability to participate in the PA intervention will not be eligible to take part in this study.”*

**Also, what if the study participants do lots of "physical activity" or exercise already, before they were enrolled in the study, would they be expected to have any effects from the intervention?**

Thank you for this feedback. We agree that those who are already engaged in a high level of physical activity would be less likely to see the benefits of the intervention. However, it is hoped that the intervention may lead to continued adherence to the PA/exercise guidelines in CF. The Fitbit will also provide the participants with the ability to self-monitor their progress. Furthermore, previous literature suggests that the majority of people with CF fail to meet their step count targets (Shelley et al., 2019).

**It is unclear why the group that also wore the fitness tracker (only) and why having people wearing the fitness tracker for 12 more weeks. Were the extra 12 weeks function like a control period? If so, would there be any carry-over effect in your experimental group if that's the case?**

The extra 12 weeks was to serve as a control period for both groups.

As this was conducted as a pilot randomised study, the authors were unsure if the Fitbit alone may lead to an increase in any of the clinical outcome measures. And if an increase might be seen at 12 weeks (in for example step count/aerobic capacity etc.), would these be sustained at 24 weeks.

**For the handgrip strength assessment, I am not sure if it really indicates "physical function." In the CF literature, handgrip strength was used as a surrogate measure for general muscle strength (Martinez-Garcia et al., 2020** **2** **; Rietschel et al., 2008** **3** **; Ward et al., 2013** **4** **). Physical function is a broader concept than muscle strength. I am curious why a study in ILD should be cited here.**

Thank you for this comment. Grip strength is a surrogate measure for general musculoskeletal strength but furthermore is correlated to lung function and VO2 peak, both of which are key indicators of prognosis in CF. The ILD study was referenced as this study looked at the test re-test reliability of a handheld dynamometer. Test re-test reliability has not been conducted in adults with CF, however there would be some similarities across cohorts in this regard.

The following has been changed in the methods section for grip strength:

*“Grip strength will be recorded as a measure of general musculoskeletal strength, independent of lower limb strength. Previous research has shown that grip strength is correlated to lung function and VO2 peak* *(Wells et al., 2014)* *. This will be conducted using a Jamar Hydraulic Hand Dynamometer. Test – retest reliability has been proven in respiratory patients (* *Dowman et al., 2016* *). The participant will be asked to stand and hold the dynamometer in by their side, with their elbow at 90 degrees and forearm in neutral. They will be instructed to squeeze the device as hard as possible. They will be provided with a 30 second rest period between each trial. The greater of two trials from each hand will be used and added together to give overall handgrip strength. This will be measured in kilograms* *(Martínez-García et al., 2020)* *.*

**Since your outcome measures were mostly self-report, or else they are easy to perform (and to learn), and there were many people working on this study, would it enhance the quality of the study by blinding the assessor (i.e. not using the same physiotherapist providing the feedback to the study participants).**

Thank you for this comment. We agree that ideally, we would prefer to use a blinded assessor, however this is not feasible in our current setting due to lack of resources.

*“Assessors cannot be blinded as the intervention involves the CF physiotherapists for the delivery of weekly text messages, who will also be assisting with repeating objective outcome measures. However, all self-report measures will be completed by the participants and these subjective assessments will be analysed by a research assistant (RA) (AJ) who is blinded to group allocation.”*

**Statistical Analysis**

**There are 10 outcome measures in this study. A p-value of p = 0.05/10 = 0.005 should be used to determine statistical significance. Perhaps the authors should consider limiting the number of outcome measures. For example, can IPAQ be eliminated since the fitness tracker was keeping track of the same/similar information? Even if only the primary outcomes are considered, the p-value of p = 0.05/2 = 0.025 should be used.**

Thank you for highlighting this. The primary outcome measure is step count and this has been adjusted in the methods section of the article. Bonferroni correction will be applied for multiple testing.

**Minor Correction Recommendation**

**In the Introduction and Abstract section, "pilot randomised trial" was written in this order, but in the Study Design section and perhaps some other places, it was put as "randomised pilot trial". To me, the former order seems correct.**

Thank you for this feedback. We agree this wording was inconsistent. We have written it as “pilot randomised trial” throughout the document.

References:

Caspersen, C. J., Powell, K. E. and Christenson, G. M. (1985) 'Physical activity, exercise, and physical fitness: definitions and distinctions for health-related research', *Public health rep,* 100(2), pp. 126-31.

Martínez-García, M. d. M., Rodríguez-Juan, J. J. and Ruiz-Cárdenas, J. D. (2020) 'Influence of sex gap on muscle strength and functional mobility in patients with cystic fibrosis', *Applied Physiology, Nutrition, and Metabolism,* 45(4), pp. 387-392.

Shelley, J., Boddy, L. M., Knowles, Z. R., Stewart, C. E. and Dawson, E. A. (2019) 'Physical activity and associations with clinical outcome measures in adults with cystic fibrosis; a systematic review', *Journal of Cystic Fibrosis*.

Tudor-Locke, C. and Bassett, D. R. (2004) 'How many steps/day are enough?', *Sports medicine,* 34(1), pp. 1-8.

Wells, G. D., Wilkes, D. L., Schneiderman, J. E., Thompson, S., Coates, A. L. and Ratjen, F. (2014) 'Physiological correlates of pulmonary function in children with cystic fibrosis', *Pediatric pulmonology,* 49(9), pp. 878-884.

# Peer review: Reviewer response for version 1

**DOI:** 10.21956/hrbopenres.14120.r27472

**Published:** 2020-06-25

**Authors:** Sarah Rand

**Affiliations:** 1 UCL Great Ormond Street Institute of Child Health, University College London, London, UK

Thank you for asking me to review this interesting and very topical protocol.

 The authors have made a very good effort at identifying the need for undertaking this study (which is even more relevant given the current circumstances around the world) for PWCF. As the authors correctly say there is an ever increasing research base investigating telehealth for individuals with long term conditions such as CF so this is a pertinent and relevant study protocol.

 Below are some specific section comments:

**Title:**

 The title is a little confusing as health is all encompassing and can include many factors from physical to mental health. Physical activity itself also includes exercise so I think that the authors perhaps need to consider making the title a little more specific to the study aims and objectives. For example is exercise going to be assessed separately to physical activity from the steps data using perhaps HR data from the FitBits? In the abstract introduction quality and wellbeing have been used as secondary 'endpoints' - is this what the authors are referring to when they say health in the title. Perhaps the title should be more specific to reflect this? Also is the aim of the study not more focussed on investigating the ability of telehealth to impact on health behaviours in PWCF? There is no mention of telehealth or technology in the title - perhaps this should be considered?

**Abstract:**

 The abstract is relatively clear however consistency with the title as suggested above is needed in terms of the aims of the study.

 The manufacturer details are needed for the Fitbit and the Fitabase to ensure reproducibility.

 What does the online monitoring system actually do - there are limited details pertaining to this? What type of data is collected and how often and therefore what type of information is available to the healthcare professional.

 I think the authors need to be clear about what the text messages will include - is it only positive reinforcement plus changes to targets based on the weekly step counts etc (how will this be progressed? Is a standardised method going to be used for all participants? References perhaps needed for this. Or will it also include 'feedback'? What will the 'feedback' include? 

 Is there a need for a wash-in period (to allow for the novelty effect) at the beginning of the study and/or a washout period between the 12 week time period to allow for the positive or negative impact of familiarisation of using a fitness tracker?

 A point of note: wearable technology is a more appropriate term rather than fitness tracker. 

**Background:**

 There is a lack of data to support the statements being made throughout this section e.g. 'higher levels of exercise' what do the authors mean by higher and using what outcome measures?

 More specific detail is required in terms of the evidence base being used to support the aims, objectives and methodology of this protocol under the 4 sub sections - CF, PA, telehealth and goal setting.

 A clear definition of physical activity is needed. Exercise is a sub section of physical activity and it is not clear if an specific 'exercise' intervention is planned via the SMS 'feedback' which should be outlined.

 In addition in the background reference to the need for an assessment of delivery and acceptability is made but this has not been mentioned in the abstract or introduction.

**Methods:**

 What do the authors mean by efficacy? Is engagement with the technology enough for the study to be deemed efficacious? Will engagement with the technology be recorded? 

 Will the outcome measures be measured during routine outpatient appointments - or will additional appointments need to be made? What will the additional burden (physical and time) be for the participants? Has there been any PPI used in the planning of this study to take this into account?

 Exclusion criteria - why have individuals with a FEV1<25% been excluded?

 The term PWCF who are pregnant rather than pregnancy should be used.

 No mention of 'usual care' has been given and would be useful for the reader to understand with respect to physical activity advice and interventions that the participants normally receive.

 Randomisation: the lung function categories are confusing - <70% mild - should this not be 70% and above? 30-50% moderate - what about those with 51-69%.....?

 Intervention - more detail is needed to clearly explain and outline all of the details pertaining to the initial information - how will this be provided - verbal or written form? What will it include? Etc.

 How will baseline physical activity be measured? Using the Fitbits provided or another accelerometer? How long will this be measured for? Specific details are needed here - number of hours and on which days - weekdays and/or weekends etc.

 Goal setting intervention - what strategies and approach will be used for this? Will a specific standardised format be used for each participant? How many goals will be used? Will this be standardised?

 As previously mentioned are wash-in and washout periods needed for data collection? Would a randomised crossover study design be more useful to account for this?

**Outcome measures:**

Primary: In the abstract PA is included as a primary endpoint but listed as a secondary outcome here.

 Is CPET an appropriate primary outcome measure - an exercise intervention has not been included or described in this protocol. Is the aim for fitness to be impacted or PA only? This needs to be clarified.

 Has the well validated supramaximal verification CPET protocol for PWCF been considered as an option?

 More details are required regarding the 'ramp protocol' that has been referred to and a reference added. What outcomes will be used from the CPET - VO2 max only or will others be considered e.g. VE/VO2, RER as well as standard CPET outcomes in terms of duration, load etc. More details are needed.

 Is FEV1 only going to be used as a classification measure? If this is the case then it is not a primary outcome measure but purely a method of categorising the participants rather than a measure of change or a measure of the impact of the technology.

 Are other PFTs measures going to be considered?

Secondary

 More detail regarding the step count data collection needed. How frequently will data be collected? How will this be analysed - how many timepoints? Will all 7 days be used or only those with a pre-specified number of hours data? Etc.

 More detail is needed regarding the protocols for each of the outcome measures e.g. hand dynamometry - will it be a best of three measure, what unit of measurement will be used, will a standardised protocol be used - what does this include?

 Will the participants complete the questionnaires independently or in the presence of a study team member?

 Why is sleep being used as an outcome measure? What is the relevance or need for this? Its not clear. This perhaps comes back to my comment regarding a need for a clear definition from the outset for 'physical activity and health'.

 There are a lot of outcome measures - again the burden to the participant needs to be considered? Has participant fatigue been considered? Are all measures necessary to answer the research question? 

 Data analysis: limited statistical analysis plans have been provided. Further details are needed and some reference to intention to treat analysis, missing data and drop outs is required. In addition it would be useful to have an idea as to the possible options for data display.

**Discussion:**

 The discussion is quite limited and a number of broad generalised statements have been made which require further detail and explanation to fully convince the reader of the rationale and need for this study.

 A clear and evidence based discussion as to the validity and reliability of the chosen wearable technology is also needed.

 A brief reference to the fact that gene therapy will be monitored during the study has been made - this is a very important point and needs to be considered carefully. A clear outline of what 'other' data will be monitored during the study is also needed - e.g. hospital admission, clinical appointments, colonisation status etc.

Is the study design appropriate for the research question?

Partly

Is the rationale for, and objectives of, the study clearly described?

Partly

Are sufficient details of the methods provided to allow replication by others?

No

Are the datasets clearly presented in a useable and accessible format?

Not applicable

Reviewer Expertise:

Cystic Fibrosis, Physical activity, exercise, health technology.

I confirm that I have read this submission and believe that I have an appropriate level of expertise to confirm that it is of an acceptable scientific standard, however I have significant reservations, as outlined above.

# Response

**Published:** 2020-09-30

**Authors:** Maire Curran

**Affiliations:** University of Limerick, Ireland

Dear Dr Sarah Rand,

**Re: Steps Ahead: Optimising physical activity in adults with cystic fibrosis: Study Protocol for a pilot randomised trial**

We sincerely thank you for your detailed and critical analysis of our article. Please find our responses below after your feedback (in bold). All changes have been made and are illustrated through italics and will be submitted in the second version of this article:

**Thank you for asking me to review this interesting and very topical protocol.**

**The authors have made a very good effort at identifying the need for undertaking this study (which is even more relevant given the current circumstances around the world) for PWCF. As the authors correctly say there is an ever-increasing research base investigating telehealth for individuals with long term conditions such as CF so this is a pertinent and relevant study protocol.**

Thank you for your positive response. We believe this is very timely research, particularly considering current circumstances and the current landscape for PWCF. We have attempted to address your concerns below to enhance the quality and transparency of our research.

**Below are some specific section comments:**

**Title:**

**The title is a little confusing as health is all encompassing and can include many factors from physical to mental health. Physical activity itself also includes exercise so I think that the authors perhaps need to consider making the title a little more specific to the study aims and objectives. For example is exercise going to be assessed separately to physical activity from the steps data using perhaps HR data from the FitBits? In the abstract introduction quality and wellbeing have been used as secondary 'endpoints' - is this what the authors are referring to when they say health in the title. Perhaps the title should be more specific to reflect this? Also is the aim of the study not more focussed on investigating the ability of telehealth to impact on health behaviours in PWCF? There is no mention of telehealth or technology in the title - perhaps this should be considered?**

Thank you for highlighting this.  We agree the title should be more specific and so this has been changed to *“Steps Ahead: optimising physical activity in Adults with Cystic Fibrosis: Study protocol for a pilot randomised trial using wearable technology”.*

Exercise is not being assessed separately to physical activity (step count data) in this present study. The Fitbit Charge 2 can measure ‘activity minutes’, through the use of heart rate (HR) data, however, this has not yet been evaluated as a valid and reliable measure for HR in people with CF. In fact, a recent study assessed HR data amongst 15 healthy participants and found that the Fitbit Charge 2 could underestimate HR by almost 30 beats per minute (Benedetto et al., 2018). Therefore, this pilot study is focusing on step count alone as a proxy measure for physical activity.

Thank you for this important observation. The use of the word health is broad and initially was used to refer to a number of variables described in the methods. As this is a pilot study, we are keen to investigate if this intervention will improve a range of clinical outcomes (including step count itself, lung function, exercise capacity, quality of life, wellbeing, and sleep). The word health in our study was used to broadly encompass these key components. However, our primary aim is to improve PA and so we have removed the word health from the title. We have provided further description of the health behaviours in the introduction and methods.

**Abstract:**

**The abstract is relatively clear however consistency with the title as suggested above is needed in terms of the aims of the study.**

**The manufacturer details are needed for the Fitbit and the Fitabase to ensure reproducibility.**

The title has changed to reflect your comments above.

Manufacturer details have been added under the title primary outcome measure – Fitbit Charge 2, as follows:

*“The Fitbit Charge 2 (Fitbit Inc.) will be used to record step count during this study with data uploaded to Fitabase (Small Steps Labs LLC).”*

**What does the online monitoring system actually do - there are limited details pertaining to this? What type of data is collected and how often and therefore what type of information is available to the healthcare professional.**

Thank you for this comment which has been addressed in the script in the intervention “wearable technology section” as follows:

*“Fitabase, the* *online monitoring system enables the physiotherapists to access step count data remotely. When a participant enables Bluetooth and syncs their Fitbit through the use of the Fitbit app, this automatically updates on the Fitabase website. This ensures that data is continuously collected over the full study duration. The data collected through the Fitbit Charge 2 and recorded on the Fitabase system are step count, activity minutes and sleep. However, for the purpose of this study, the researchers are only investigating step count alone. Any further research would require the Fitbit to be assessed under each of these conditions to ensure validity and reliability. This is outside the scope of this research.”*

**I think the authors need to be clear about what the text messages will include - is it only positive reinforcement plus changes to targets based on the weekly step counts etc (how will this be progressed? Is a standardised method going to be used for all participants? References perhaps needed for this. Or will it also include 'feedback'? What will the 'feedback' include?** 

Thank you for this feedback. They following text has been added to the intervention section to ensure clarity around text messaging.

*“The text messages in this study will be personalised, one-way texts with positive reinforcement on step count attained by the participant.*

*A metanalysis found personalisation strategies such as using a participant name has been proven to increase intervention efficacy* *(Head et al., 2013)* *and tailoring messages is a key ingredient in text messaging interventions* *(Noar et al., 2007)* *. Furthermore, one way communication is as effective as two-way dialogue* *(Head et al., 2013)* *. Therefore, this study investigated one-way messages only.*

*An individualised message will be sent to each participant once a week, at a day and time chosen by the participant at the start of the intervention. The authors will follow a standardised approach. The message will address the participant by name and will have positive feedback* *in the form of encouragement and praise messages* *(Adams et al., 2013)* *in relation to their step count attained in the previous week. It will then focus on a physical activity related goal set by the subject and will sign off with a further positive comment.  Research has indicated that it is crucial to reinforce improvements to develop new behaviour or to strengthen a habit* *(Hovell et al., 2009, Glanz et al., 2008, Bandura, 2004)* *.* *Each time a participant meets his/her goal they will receive positive feedback. Participants who do not meet the goal will be provided with a positive comment in relation to their step count achieved to date and provided their next step goal. This is to avoid negative messages that could be discouraging* *(Adams et al., 2013)* *.”*

**Is there a need for a wash-in period (to allow for the novelty effect) at the beginning of the study and/or a washout period between the 12 week time period to allow for the positive or negative impact of familiarisation of using a fitness tracker?**

The authors acknowledge that no wash-in or wash-out period was applied and accept that there may be a novelty effect at the start of the study. This study included public and patient involvement (PPI) at the methodological development stage and both participants and clinicians felt that a 12-week intervention would be more readily accepted by this cohort as the study appointments could coincide with their routine clinic appointments. However, data will be regularly uploaded to Fitabase and therefore we can analyse and determine if there is a significant increase in steps in the first two weeks in comparison to subsequent weeks per participant and as a group.

**A point of note: wearable technology is a more appropriate term rather than fitness tracker.**

Thank you for this comment. We agree with this and the term wearable technology has been used throughout the article.

**Background:**

**There is a lack of data to support the statements being made throughout this section e.g. 'higher levels of exercise' what do the authors mean by higher and using what outcome measures?**

**More specific detail is required in terms of the evidence base being used to support the aims, objectives and methodology of this protocol under the 4 sub sections - CF, PA, telehealth and goal setting.** **A clear definition of physical activity is needed. Exercise is a sub section of physical activity and it is not clear if an specific 'exercise' intervention is planned via the SMS 'feedback' which should be outlined.**

The authors thank the reviewer for this comprehensive feedback. A definition of PA has been added to the introduction along with further evidence to support PA in CF. There is no specific ‘exercise’ intervention planned via the feedback provided through the text messages. The study aims to look at step count behaviour only. The statements around ‘higher levels of exercise’ has been re-phrased. The following has been added/modified to the introduction:

*“PA can be described as any bodily movement that causes an increase in energy expenditure above that of resting energy expenditure* *including leisure-time PA, occupational PA and exercise* *(Caspersen et al., 1985)* *. Exercise and physical activity (PA) are widely documented in consensus statements as key components in the management of CF (* *Castellani et al., 2018* *). PA has several positive benefits in this population as it has been shown to improve sputum clearance (* *Dwyer et al., 2009* *), bone mineral density* *(Gupta et al., 2019)* *and muscle strength (* *Burtin et al., 2013* *). More importantly, PA can improve aerobic capacity* *(Hebestreit et al., 2010)* *and it can slow the rate of decline in lung function* *(Schneiderman et al., 2014)* *, both of which are linked to increased survival in CF* *(Nixon et al., 1992)* *. As a result, the optimisation of exercise and PA among PWCF is important, however, a Cochrane review found that there was a lack of evidence regarding strategies to promote PA in this population and consideration should be given towards telemedicine applications and health coaching (* *Cox et al., 2013b* *).*

*Numerous subjective and objective methods are reported in the literature for the assessment of PA in adults with CF. Step count measurement can be used to quantify and monitor PA behaviours* *(Tudor-Locke and Bassett, 2004)* *. Despite the clear benefits of PA in CF, a recent systematic review found that adults with CF fail to meet recommended PA and step count guidelines* *(Shelley et al., 2019)* *. Therefore, interventions which aim to increase PA in this population are of considerable interest.*

*In addition to telehealth, goal setting and reviewing goals regularly should be considered to support patients with chronic illness (* *Coleman & Newton, 2005* *). In order for goals to be achieved, feedback should be provided that reveals progress in relation to the goal (* *Locke & Latham, 2002* *). Furthermore, setting specific goals should offer a plan to break PA goals into more practical, manageable steps (* *Shilts et al., 2004* *) which should increase self-efficacy and hence promote continued regular PA levels (* *Bandura, 2004* *). Previous literature from patient preference research and effective PA promotion approaches reports that successful strategies include specifically targeting PA* *(Conn et al., 2008)* *, the use of behavioural strategies such as feedback and goal setting* *(Conn et al., 2008, Kosma et al., 2005)* *(George et al., 2012)* *and the ability to self-monitor* *(Conn et al., 2008)* *. The Irish national framework for self-management includes goal setting and action planning in chronic disease management (* *Chronic Conditions Working Group, 2017* *). PA interventions using pedometers have been shown to be more effective at increasing PA if they include step goals (Bravata et al., 2007). However, goal setting to increase PA in adults with CF is poorly investigated.”*

**In addition in the background reference to the need for an assessment of delivery and acceptability is made but this has not been mentioned in the abstract or introduction.**

Thank you for highlighting this. The following has now been added to the introduction:

*“This pilot randomised trial aims to explore the impact of wearable technology, text message personalised feedback and goal setting on PA and health* *outcomes* *in adults with CF. For the purpose of this study these specific health* *outcomes* *include lung function, aerobic capacity, body composition, quality of life, well-being and sleep. Poor sleep quality has been previously highlighted as an issue in adults with CF* *(Milross et al., 2002)* *and PA interventions can improve sleep quality in other populations* *(Yang et al., 2012, Lan et al., 2014)* *.*

*In addition, it is important to conduct a qualitative analysis to evaluate the implementation, delivery and acceptability of the intervention as this is the key to the development of future research which this pilot study aims to inform. Therefore, semi structured interviews will be conducted with the study participants and with the healthcare professionals providing the intervention. This will aim to inform future studies in this evolving research area.”*

**Methods:**

**What do the authors mean by efficacy? Is engagement with the technology enough for the study to be deemed efficacious? Will engagement with the technology be recorded?**

**The authors are referring to the ability of the tracker to change PA levels, FEV1 or exercise capacity (being the primary outcome measures).**

Thank you for your feedback on this. The term efficacy is nebulous and so this has been changed to more concrete term reflecting what we aim to do in this study -

*“This study represents a single centre pilot randomised trial which will compare the effect of wearable technology with personalised text message feedback and goal setting to wearable technology alone in adults CF.”*

Efficacy refers to the ability of the wearable technology, text message feedback and goal setting to increase PA levels (being the primary measure). The following has been added in relation to engagement with the technology:

*“Engagement with the technology will be monitored through systematic review of online data. Participants in both groups will receive a “reminder” message on his or her mobile phone if their data has not been synced via the Fitbit App in the previous seven days. This ensures that data is continuously collected over the full study duration.”*

**Will the outcome measures be measured during routine outpatient appointments - or will additional appointments need to be made? What will the additional burden (physical and time) be for the participants? Has there been any PPI used in the planning of this study to take this into account?**

Thank you for highlighting this. We have added the following for clarification on appointments -

*“E *ach adult with CF attends their routine clinic appointments every three months. Therefore, we chose this duration for our intervention to dovetail with routine CF care and limit the need for participants to attend on a more regular basis for testing appointments. This was decided through PPI (completed with adults with CF) and with clinician input. If the participant cannot attend on a particular clinic day, then they will be offered an additional appointment to suit their schedule.”**

*The cardiopulmonary exercise test (CPET) is only completed at annual assessment typically, and so, this will require additional time on their routine clinic appointment. It is aimed to conduct this CPET either pre or post clinic review. The anticipated time to complete the CPET is between eight to 12 minutes.*

**Exclusion criteria - why have individuals with a FEV1<25% been excluded?**

The following has been added to the exclusion section:

*“Individuals with FEV _1_ <25% would typically require supplemental oxygen for exercise and at this point may require transplant assessment. Furthermore, they are more likely to experience exacerbations and would not be suitable for a study over 24 weeks. This is a lower cutoff FEV _1_ than most studies would include* *(Kriemler et al., 2013, Hebestreit et al., 2010, Klijn et al., 2004)* *”*

**The term PWCF who are pregnant rather than pregnancy should be used.**

Thank you, this has been changed in the manuscript.

**No mention of 'usual care' has been given and would be useful for the reader to understand with respect to physical activity advice and interventions that the participants normally receive.**

Thank you for this suggestion which the authors agree would be helpful to include in the manuscript. We have added the following to the methods section of the article:

*“Usual care would typically include PA and exercise advice as per Australian and New Zealand Cystic Fibrosis guidelines* *(Button et al., 2016)* *. People with CF are advised to exercise for at least 30 minutes, five days a week. They are advised to include a combination of aerobic and resistance training. It is personalised depending on patient preference* *(Button et al., 2016)* *.”*

**Randomisation: the lung function categories are confusing - <70% mild - should this not be 70% and above? 30-50% moderate - what about those with 51-69%.....?**

Thank you for this comment. This was an oversight and has been adjusted as per below.

*“* *Following this a minimisation randomisation procedure will be completed based on lung function, where FEV _1_ of >80% predicted lung function will be classified as having mild lung disease. While those with an FEV _1_ of 50-79% predicted lung function will be classified as having moderate lung disease, 30-49% as severe lung disease and <30% indicating severe lung disease* *(Ranu et al., 2011)* *”*

**Intervention - more detail is needed to clearly explain and outline all of the details pertaining to the initial information - how will this be provided - verbal or written form? What will it include? Etc.**

We have endeavoured to provide more detail on the intervention in the manuscript as follows:

*“Baseline activity levels will be obtained using an ActivPAL accelerometer as part of a parallel study which assesses PA levels in this group. This will be used to inform goal setting only. The data obtained from the ActivPAL will be discussed with the participant at baseline.  D* *ata will be included if at least four full days (at least three weekdays and one weekend day) of measurements with a minimum of 10 hours (h) for the weekdays and 10 h for the weekends* *are measured* *(Freedson et al., 2005)* *.* **Based on PPI it was reported by the participants that they did not want to wear an ActivPAL other than at baseline as it is a thigh worn device which is not ‘fashionable’. Similar concerns have been cited previously in the research** *(Dias et al., 2012)* *.*

*“The intervention consists of wearable technology, text message feedback and goal setting.*

*Wearable technology:*

*If the participant is allocated to the intervention, they will be provided with wearable technology (Fitbit Charge 2), educated on how to use it, and this will also be linked to an online monitoring system (Fitabase). Participants will be encouraged to enable Bluetooth and upload data regularly.*

*Goal setting:*

*The physiotherapist will discuss the participant’s PA levels (as measured at baseline by an accelerometer) and individual patient centred PA goals will be set with each participant.* *These will be discussed with their physiotherapist to ensure they are specific, measurable, achievable, realistic and timed (SMART). They will be encouraged to write a minimum of three goals. Participants will be asked to set a step count target for week four, eight and 12 and consider ways to achieve these goals, both of which will be discussed with the physiotherapist during the baseline appointment. Goals will be individualised to the participant taking into account their preferences. Participants may also set goals related to health outcomes being assessed – for example, a participant may have a goal to improve lung function, aerobic capacity, sleep etc. during the study period. The text message feedback will refer to step goals only.  The participant will be given a copy of their goals.*

*Text message feedback:*

*The text messages in this study will be personalised, one-way texts with positive reinforcement on step count attained by the participant.* *A metanalysis found personalisation strategies such as using a participant name has been proven to increase intervention efficacy* *(Head et al., 2013)* *and tailoring messages is a key ingredient in text messaging interventions* *(Noar et al., 2007)* *. Furthermore, one way communication is as effective as two-way dialogue* *(Head et al., 2013)* *. Therefore, this study investigated one-way messages only.*

*An individualised message will be sent to each participant once a week, at a day and time chosen by the participant at the start of the intervention. The authors will follow a standardised approach. The message will address the participant by name and will have positive feedback* *in the form of encouragement and praise messages* *(Adams et al., 2013)* *in relation to their step count attained in the previous week. It will then focus on a physical activity related goal set by the subject and will sign off with a further positive comment.  Research has indicated that it is crucial to reinforce improvements to develop new behaviour or to strengthen a habit* *(Hovell et al., 2009, Glanz et al., 2008, Bandura, 2004)* *.* *Each time a participant meets his/her goal they will receive positive feedback. Participants who do not meet the goal will be provided with a positive comment in relation to their step count achieved to date and provided their next step goal. This is to avoid negative messages that could be discouraging* *(Adams et al., 2013)* *.* *If the participant does not achieve their target step count goal then the physiotherapist will set a target increase of 10% from the mean step count achieved in the previous week.*

*A sample text message is as follows:*

*Hi Paul, Well done on achieving an average daily step count of 7,600 steps this week. Next week aim to hit your goal of 8,000 steps. Keep up the good work!*

**How will baseline physical activity be measured? Using the Fitbits provided or another accelerometer? How long will this be measured for? Specific details are needed here - number of hours and on which days - weekdays and/or weekends etc.**

As per above previous comment above, baseline PA levels will be measured by an ActivPAL accelerometer (PAL technologies). This will not be further used in the study as based on PPI adults with CF will not comply to wear this device on subsequent testing. This has been added to the methods section of the article.

**Goal setting intervention - what strategies and approach will be used for this? Will a specific standardised format be used for each participant? How many goals will be used? Will this be standardised?**

Please see previous paragraph on goal setting. This will be illustrated under the Intervention section of the article.

**As previously mentioned are wash-in and washout periods needed for data collection? Would a randomised crossover study design be more useful to account for this?**

As mentioned above we felt that a wash-in and wash out period would not be necessary in a small pilot study that is 12 weeks in duration with a further 12 week follow up. A crossover study would increase the need for a washout period and would prolong the length of the study. We had considered a crossover design but we felt this was too complex for a small pilot study and taking into account overall patient burden.

**Outcome measures:**

**Primary: In the abstract PA is included as a primary endpoint but listed as a secondary outcome here**

Thank you for highlighting this. This has been changed. It is a primary outcome measure.

**Is CPET an appropriate primary outcome measure - an exercise intervention has not been included or described in this protocol. Is the aim for fitness to be impacted or PA only? This needs to be clarified.**

Thank you for this feedback. CPET is a secondary outcome measure.

**Has the well validated supramaximal verification CPET protocol for PWCF been considered as an option?**

Thank you for this suggestion. The supramaximal verification protocol will be used. This has been added to the methods section of the study.**

*“Supramaximal verification be conducted to ensure a maximal effort during the CPET.* *After an incremental CPET, participants will be provided with a ten-minute break. They will then be asked to complete a supramaximal exercise test* *(Causer et al., 2018, Saynor et al., 2013)* *whereby participants will exercise at 110% of their power output (as determined by CPET test previously). This test has been conducted amongst PWCF previously and has been deemed safe and ensures validity of the results which are obtained in a CPET test.”*

**More details are required regarding the 'ramp protocol' that has been referred to and a reference added. What outcomes will be used from the CPET - VO2 max only or will others be considered e.g. VE/VO2, RER as well as standard CPET outcomes in terms of duration, load etc. More details are needed.**

Thank you for this comment. The following text has been added to the methods section under secondary outcome measures and “CPET”.

*“The reference standard exercise test is an incremental cardiopulmonary exercise test (CPET), utilising a ramp protocol* *. The ramp protocol ensures that work rate is progressively and linearly incremented until maximal effort is achieved* *(Herdy et al., 2016)* *.* *All standard CPET outcomes will be considered including duration, load, pulmonary ventilation (VE), Respiratory Exchange Ratio (RER), ventilatory equivalents for oxygen (VE/VO _2_) and for carbon dioxide (VE/VCO _2_).”*

**Is FEV1 only going to be used as a classification measure? If this is the case then it is not a primary outcome measure but purely a method of categorising the participants rather than a measure of change or a measure of the impact of the technology.**

**Are other PFTs measures going to be considered?**

Thank you for highlighting this. FEV _1_ will be used as a classification measure but will also be used to determine the effect that this intervention may have on FEV _1_ as a secondary outcome measure. Furthermore, we will also consider other PFT’s such as FVC and FEF25-75. The following has been added to the methods section:

*“Forced expiratory volume in one second (FEV _1_) will be used to classify the severity of CF lung disease for each participant. It will also be used to determine the effect the intervention may have on pulmonary function. *Other pulmonary function measures such as % forced vital capacity (FVC) and forced expiratory flow (FEF25-75) will also be considered.”**

**Secondary**

**More detail regarding the step count data collection needed. How frequently will data be collected? How will this be analysed - how many timepoints? Will all 7 days be used or only those with a pre-specified number of hours data? Etc.**

The data will be continuously collected throughout the study time period.

Every time the participant syncs their Fitbit, the data will automatically upload on to Fitabase.

The following has been added to the data analysis section: 

*“Data will be collected over seven days and an average weekly step count will be obtained.  It is anticipated that step count data will be analysed to assess for a novelty effect in the first two weeks, at week six and 12 during the intervention and at week 18 and Week 24 for the follow up. A repeated measures ANOVA, controlling for baseline values and other potential confounders will be conducted.”*

**More detail is needed regarding the protocols for each of the outcome measures e.g. hand dynamometry - will it be a best of three measure, what unit of measurement will be used, will a standardised protocol be used - what does this include?**

Thank you for this feedback. We have added more detail to the protocol in the methods section. **

*“* *The participant will be asked to stand and hold the dynamometer in by their side, with their elbow at 90 degrees and forearm in neutral. They will be instructed to squeeze the device as hard as possible. They will be provided with a 30 second rest period between each trial. The greater of two trials from each hand will be used and added together to give overall handgrip strength. This will be measured in kilograms* *(Martínez-García et al., 2020)* *.*

**Will the participants complete the questionnaires independently or in the presence of a study team member?**

Thank you for highlighting this. The following has been added to the Data Collection and Management section:

*“All self-report measures will be completed by the participants independently,* *however a* *research assistant* *(RA) (AJ)* *will be available if there are any queries or concerns.* *These subjective assessments will be analysed by the RA who is blinded to group allocation.”*

**Why is sleep being used as an outcome measure? What is the relevance or need for this? It’s not clear. This perhaps comes back to my comment regarding a need for a clear definition from the outset for 'physical activity and health'.**

Thank you for this suggestion. We have added the following to clarify these specific health outcomes in the introduction section:

*“This pilot randomised trial aims to explore the impact of wearable technology, text message personalised feedback and goal setting on PA and health* *outcomes* *in adults with CF. For the purpose of this study these specific health* *behaviours* *include lung function, aerobic capacity, quality of life, well-being and sleep. Poor sleep quality has been previously highlighted as an issue in adults with CF* *(Milross et al., 2002)* *and PA interventions can improve sleep quality in other populations* *(Yang et al., 2012, Lan et al., 2014)* *.”*

**There are a lot of outcome measures - again the burden to the participant needs to be considered? Has participant fatigue been considered? Are all measures necessary to answer the research question?** 

Participant fatigue has been considered. The authors have used a PPI approach in the study design and set up and based on feedback participants were satisfied with timing and breaks between each outcome assessment. Furthermore, the adults with CF attending this CF centre would be familiar with most of these outcome measures which would be conducted as part of their annual assessment. As this is a pilot study the authors incorporated a range of outcome measures to answer the key research questions and to inform future research.

**Data analysis: limited statistical analysis plans have been provided. Further details are needed and some reference to intention to treat analysis, missing data and drop outs is required. In addition it would be useful to have an idea as to the possible options for data display.**

The following has been added to the data analysis section:

*“Primary analyses will be performed according to intention-to-treat (ITT) principles with all participants who were originally allocated by randomisation and those who dropped out from the study. In case of missing values, a multiple imputation method, inverse probability weighing or mixed models will be used to handle the missing data.* *Data will be presented as means and SDs for data that is normalised and medians (IQR) for data that is skewed.* *”*

**Discussion:**

**The discussion is quite limited and a number of broad generalised statements have been made which require further detail and explanation to fully convince the reader of the rationale and need for this study.**

The discussion has been revised with further detail added on the rationale and need for the study.

**A clear and evidence based discussion as to the validity and reliability of the chosen wearable technology is also needed.**

Thank you for highlighting this. This research has been conducted and has been submitted for publication. This research concluded that Fitbit Charge 2 ranged from weak to very strong correlations when compared to visual observation (0.34-0.84). The Fitbit Charge 2 underestimated step count by 2.8%-9.2%. This is within acceptable limits of variability based on previous research (Schneider et al., 2004). The following has been added to the methods:

*“Prior to this intervention, the validity and reliability of the Fitbit Charge 2 to measure step count in CF was assessed by the same research group* *(Curran et al., 2020)* *. Twenty-one participants were recruited from an adult CF Centre for a single session of testing. Participants walked for five minutes at five pre-determined speeds in a controlled testing environment (2, 2.5, 3, 3.5 and 4 miles per hour on a treadmill) and at three self-selected speeds on a corridor (slow, medium and fast). The Fitbit Charge 2 was compared to visual observation. It was found that the Fitbit Charge 2 ranged from weak to very strong correlations when compared to visual observation (0.34-0.84). The Fitbit Charge 2 underestimated step count by 2.8%-9.2%. This is within acceptable limits of variability based on previous research* *(Schneider et al., 2004)* *.”*

**A brief reference to the fact that gene therapy will be monitored during the study has been made - this is a very important point and needs to be considered carefully. A clear outline of what 'other' data will be monitored during the study is also needed - e.g. hospital admission, clinical appointments, colonisation status etc.**

Thank you for this comment.

*“The research team will also monitor *exacerbation rates (participants requiring oral or IV antibiotics during the study period), hospital admission rate and colonisation status.”**

References:

Adams, M. A., Sallis, J. F., Norman, G. J., Hovell, M. F., Hekler, E. B. and Perata, E. (2013) 'An adaptive physical activity intervention for overweight adults: a randomized controlled trial', *PloS one,* 8(12), pp. e82901.

Bandura, A. (2004) 'Health promotion by social cognitive means', *Health Education & Behavior,* 31(2), pp. 143-164.

Benedetto, S., Caldato, C., Bazzan, E., Greenwood, D. C., Pensabene, V. and Actis, P. (2018) 'Assessment of the Fitbit Charge 2 for monitoring heart rate', *PloS one,* 13(2), pp. e0192691.

Button, B. M., Wilson, C., Dentice, R., Cox, N. S., Middleton, A., Tannenbaum, E., Bishop, J., Cobb, R.,

Burton, K. and Wood, M. (2016) 'Physiotherapy for cystic fibrosis in Australia and New Zealand: a clinical practice guideline', *Respirology,* 21(4), pp. 656-667.

Caspersen, C. J., Powell, K. E. and Christenson, G. M. (1985) 'Physical activity, exercise, and physical fitness: definitions and distinctions for health-related research', *Public health rep,* 100(2), pp. 126-31.

Causer, A. J., Shute, J. K., Cummings, M. H., Shepherd, A. I., Bright, V., Connett, G., Allenby, M. I.,

Carroll, M. P., Daniels, T. and Saynor, Z. L. (2018) 'Cardiopulmonary exercise testing with supramaximal verification produces a safe and valid assessment of VO2max in people with cystic fibrosis: a retrospective analysis', *Journal of Applied Physiology,* 125(4), pp. 1277-1283.

Conn, V. S., Hafdahl, A. R., Brown, S. A. and Brown, L. M. (2008) 'Meta-analysis of patient education interventions to increase physical activity among chronically ill adults', *Patient education and counseling,* 70(2), pp. 157-172.

Curran, M., Tierney, A., Collins, L., Kennedy, L., McDonnell, C., Sheikhi, A., Walsh, C., Casserly, B. and Cahalan, R. (2020) 'Validity and Reliability of the ActivPAL and Fitbit Charge 2 as a measure of step count in Cystic Fibrosis', *Under Review*.

Dias, A., Gorzelniak, L., Jörres, R. A., Fischer, R., Hartvigsen, G. and Horsch, A. (2012) 'Assessing physical activity in the daily life of cystic fibrosis patients', *Pervasive and Mobile Computing,* 8(6), pp. 837-844.

Freedson, P., Pober, D. and Janz, K. F. (2005) 'Calibration of accelerometer output for children', *Medicine & Science in Sports & Exercise,* 37(11), pp. S523-S530.

George, E. S., Kolt, G. S., Duncan, M. J., Caperchione, C. M., Mummery, W. K., Vandelanotte, C., Taylor, P. and Noakes, M. (2012) 'A review of the effectiveness of physical activity interventions for adult males', *Sports Medicine,* 42(4), pp. 281-300.

Glanz, K., Rimer, B. K. and Viswanath, K. (2008) *Health behavior and health education: theory, research, and practice.* John Wiley & Sons.

Gupta, S., Mukherjee, A., Lodha, R., Kabra, M., Deepak, K. K., Khadgawat, R., Talwar, A. and Kabra, S. K. (2019) 'Effects of exercise intervention program on bone mineral accretion in children and adolescents with cystic fibrosis: a randomized controlled trial', *The Indian Journal of Pediatrics,* 86(11), pp. 987-994.

Head, K. J., Noar, S. M., Iannarino, N. T. and Harrington, N. G. (2013) 'Efficacy of text messaging-based interventions for health promotion: a meta-analysis', *Social science & medicine,* 97, pp. 41-48.

Hebestreit, H., Kieser, S., Junge, S., Ballmann, M., Hebestreit, A., Schindler, C., Schenk, T., Posselt, H. and Kriemler, S. (2010) 'Long-term effects of a partially supervised conditioning programme in cystic fibrosis', *European Respiratory Journal,* 35(3), pp. 578-583.

Herdy, A. H., Ritt, L. E. F., Stein, R., Araújo, C. G. S. d., Milani, M., Meneghelo, R. S., Ferraz, A. S., Hossri, C., Almeida, A. E. M. d. and Fernandes-Silva, M. M. (2016) 'Cardiopulmonary exercise test: background, applicability and interpretation', *Arquivos brasileiros de cardiologia,* 107(5), pp. 467-481.

Hovell, M., Wahlgren, D. and Adams, M. (2009) 'The logical and empirical basis for the behavioral ecological model', *Emerging theories in health promotion practice and research,* 2, pp. 347-385.

Klijn, P. H., Oudshoorn, A., van der Ent, C. K., van der Net, J., Kimpen, J. L. and Helders, P. J. (2004) 'Effects of anaerobic training in children with cystic fibrosis: a randomized controlled study', *Chest,* 125(4), pp. 1299-1305.

Kosma, M., Cardinal, B. J. and McCubbin, J. A. (2005) 'A pilot study of a web-based physical activity motivational program for adults with physical disabilities', *Disability and rehabilitation,* 27(23), pp. 1435-1442.

Kriemler, S., Kieser, S., Junge, S., Ballmann, M., Hebestreit, A., Schindler, C., Stüssi, C. and Hebestreit, H. (2013) 'Effect of supervised training on FEV1 in cystic fibrosis: a randomised controlled trial', *Journal of Cystic Fibrosis,* 12(6), pp. 714-720.

Lan, C.-C., Huang, H.-C., Yang, M.-C., Lee, C.-H., Huang, C.-Y. and Wu, Y.-K. (2014) 'Pulmonary rehabilitation improves subjective sleep quality in COPD', *Respiratory care,* 59(10), pp. 1569-1576.

Martínez-García, M. d. M., Rodríguez-Juan, J. J. and Ruiz-Cárdenas, J. D. (2020) 'Influence of sex gap on muscle strength and functional mobility in patients with cystic fibrosis', *Applied Physiology, Nutrition, and Metabolism,* 45(4), pp. 387-392.

Milross, M. A., Piper, A. J., Norman, M., Dobbin, C. J., Grunstein, R. R., Sullivan, C. E. and Bye, P. T. (2002) 'Subjective sleep quality in cystic fibrosis', *Sleep Medicine,* 3(3), pp. 205-212.

Nixon, P. A., Orenstein, D. M., Kelsey, S. F. and Doershuk, C. F. (1992) 'The prognostic value of exercise testing in patients with cystic fibrosis', *New England Journal of Medicine,* 327(25), pp. 1785-1788.

Noar, S. M., Benac, C. N. and Harris, M. S. (2007) 'Does tailoring matter? Meta-analytic review of tailored print health behavior change interventions', *Psychological bulletin,* 133(4), pp. 673.

Ranu, H., Wilde, M. and Madden, B. (2011) 'Pulmonary function tests', *The Ulster medical journal,* 80(2), pp. 84.

Saynor, Z. L., Barker, A. R., Oades, P. J. and Williams, C. A. (2013) 'A protocol to determine valid V˙ O2max in young cystic fibrosis patients', *Journal of Science and Medicine in Sport,* 16(6), pp. 539-544.

Schneider, P. L., Crouter, S. E. and Bassett, D. R. (2004) 'Pedometer measures of free-living physical activity: comparison of 13 models', *Medicine and Science in Sports and Exercise,* 36(2), pp. 331-335.

Schneiderman, J. E., Wilkes, D. L., Atenafu, E. G., Nguyen, T., Wells, G. D., Alarie, N., Tullis, E., Lands, L. C., Coates, A. L., Corey, M. and Ratjen, F. (2014) 'Longitudinal relationship between physical activity and lung health in patients with cystic fibrosis', *European Respiratory Journal,* 43(3), pp. 817-823.

Shelley, J., Boddy, L. M., Knowles, Z. R., Stewart, C. E. and Dawson, E. A. (2019) 'Physical activity and associations with clinical outcome measures in adults with cystic fibrosis; a systematic review', *Journal of Cystic Fibrosis*.

Tudor-Locke, C. and Bassett, D. R. (2004) 'How many steps/day are enough?', *Sports medicine,* 34(1), pp. 1-8.

Yang, P.-Y., Ho, K.-H., Chen, H.-C. and Chien, M.-Y. (2012) 'Exercise training improves sleep quality in middle-aged and older adults with sleep problems: a systematic review', *Journal of physiotherapy,* 58(3), pp. 157-163.
